# Probing Reconstituted Human Immune Systems in Mice With Oncogenic γ-Herpesvirus Infections

**DOI:** 10.3389/fimmu.2020.581419

**Published:** 2020-09-09

**Authors:** Christian Münz

**Affiliations:** Viral Immunobiology, Institute of Experimental Immunology, Zurich, Switzerland

**Keywords:** Epstein Barr virus, Kaposi sarcoma associated herpesvirus, primary effusions lymphoma, diffuse large B cell lymphoma, T cells, NK cells, NKT cells

## Abstract

Mice with reconstituted human immune systems can mount cell-mediated immune responses against the human tumor viruses Epstein Barr virus (EBV) and Kaposi sarcoma associated herpesvirus (KSHV). Primarily cytotoxic lymphocytes protect the vast majority of persistently infected carriers of these tumor viruses from the respective malignancies for life. Thus, EBV and KSHV infection can teach us how this potent immune control is induced, what phenotype and functions characterize the protective lymphocyte compartments and if similar immune responses could be induced by vaccination. This review will summarize similarities and differences between EBV and KSHV associated pathologies and their immune control in patients and mice with reconstituted human immune systems. Furthermore, it will high-light which aspects of the near perfect immune control can be modeled in the latter preclinical animal models and discuss their relevance for cancer immunology in general.

## Introduction on EBV and KSHV Specific Immune Control

The two human γ-herpesviruses Epstein Barr virus (EBV) and Kaposi sarcoma associated herpesvirus (KSHV) are WHO class I carcinogens (1–3). They are associated with lymphomas, Hodgkin’s and Burkitt’s in the case of EBV, and primary effusion lymphoma (PEL), and multicentric Castleman’s disease (MCD) for KSHV (4). Furthermore, EBV causes epithelial cell tumors, like nasopharyngeal and 10% of gastric carcinoma (5), while KSHV is the etiological cause of the endothelial cell derived cancer Kaposi sarcoma (3). Fortunately, these malignancies are rare with incidence rates of less than 1 in 10^4^ individuals, even so more than 95% of the human adult population is persistently infected with EBV, and more than 50% of the Sub-Saharan African population with KSHV (3, 6). However, EBV and KSHV associated lymphomas as well as Kaposi sarcoma develop at increased frequencies after immune suppression, due to for example human immunodeficiency virus (HIV) infection or iatrogenic treatment after transplantation (7, 8). Moreover, primary immunodeficiencies, often caused by individual gene mutations, can predispose for EBV and KSHV associated diseases (9). Interestingly, the affected genes point toward well defined immune compartments that control EBV and KSHV infection and prevent associated malignancies in most virus carriers. Even so EBV and KSHV are closely related γ-herpesviruses, the requirements for their immune control seem to be quite dissimilar. Primary immunodeficiencies that predispose for EBV associated diseases affect the cytotoxic machinery of lymphocytes, including natural killer (NK) and CD8^+^ T cells (10, 11). In addition, co-stimulatory molecules on these cytotoxic lymphocytes, like CD27, 4-1BB, 2B4, and NKG2D, seem to be required to control EBV. A third group of molecules that are required for EBV specific immune control, are involved in T cell receptor signaling, such as interleukin-2 inducible T cell kinase (ITK), ZAP70, and PI3K. Finally, gene products that support and sustain cytotoxic lymphocyte development and expansions, like MCM4, CTPS1, and XIAP, are required for EBV specific immune control. Surprisingly, type I and II interferons (IFNs) as well as antibody responses are dispensable for the immune control of EBV (11). In contrast, type II IFN signaling with IFN-γ receptor 1 and STAT4 protects from Kaposi sarcoma (9, 12). In addition, OX40 instead of the above mentioned co-stimulatory molecules is required to protect from Kaposi sarcoma (13). Thus, primary immunodeficiencies suggest that cytotoxic lymphocytes keep persistent EBV infection in check, while their cytokine production protects in addition from KSHV. Accordingly, these two pathogens are suitable to challenge human immune systems with their tumorigenic potential and test their robustness in raising cell-mediated immune responses.

## Virus Associated Malignancies

**FIGURE 1:**
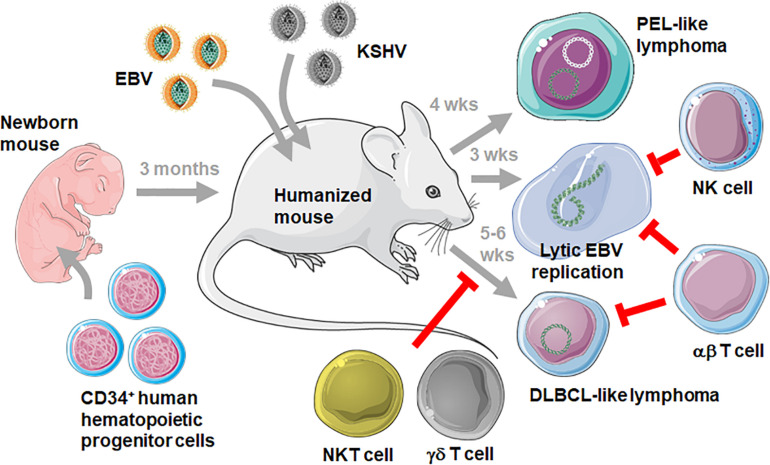
Challenging humanized mice with EBV and KSHV infection. Mice with reconstituted human immune system compartments (humanized mice) are generated by injecting human CD34^+^ HPCs into immune compromised mice, such as the NOD-*scid* γ_*c*_^– /–^ mouse strain. After 3 months humanized mice can be infected with EBV with and without KSHV. Persistent infection with these two viruses develops into PEL-like lymphomas after double infection after 4 weeks (wks), EBV lytic replication can be observed after 3 weeks and DLBCL-like lymphomas develop after 5–6 weeks. While the immune control of KSHV in humanized mice still needs to be defined, NK and αβ T cells control lytic EBV replication, and NKT, γδ T and αβ T cells prevent DLBCL-like tumorigenesis by targeting latent EBV infection. This figure was created in part with modified Servier Medical Art templates, which are licensed under a Creative Commons Attribution 3.0 unported license: https://smart.servier.com.

Such challenges are for example applied to immune compromised mice with reconstituted human immune system components, such as NOD-*scid* γ_*c*_^–/–^ that are neonatally injected with human CD34^+^ hematopoietic progenitor cells (HPCs) and then develop human leucocytes during three to 6 months (humanized mice) (14, 15) (Figure 1). In order, however, to explore the protective capacity of the reconstituted human immune system compartments, at least some of the EBV and KSHV associated pathologies must be recapitulated in these humanized mouse systems. Accordingly, the viral gene expression patterns have been analyzed after EBV infection of humanized mice (16–21). In most of these studies a high dose of infectious viral particles from the B95-8 EBV strain, originally isolated during symptomatic primary infection called infectious mononucleosis (IM), was used. As in secondary lymphoid tissues, including tonsils, of IM patients (22) primarily EBV infected B cells expressing the latency III infection program with six nuclear antigen (EBNA1, 2, 3A-C, and LP), two latent membrane proteins (LMP1,2) as well as non-translated EBER and viral miRNAs, were observed (21). In addition, however, low levels of latency I and II with EBNA1 as the sole or in addition LMP1 and 2 protein expression could be detected (18, 23). Low levels of these lower latencies were also verified by detection of characteristic viral transcripts (21, 24, 25). While latency III is found in naïve B cells of healthy EBV carriers, latency II predominates in germinal center B cells and latency I in homeostatically replicating memory B cells (26, 27). It is unclear if the respective latencies are also in similar B cell differentiation stages in humanized mice. In humans, EBV persists without viral protein expression in circulating memory B cells of so-called latency 0 (28). This latency 0 can also be reached in humanized mice (20). From latency 0 and I EBV switches into lytic replication upon plasma cell differentiation in healthy virus carriers (29). Such reactivation only rarely occurs with the B95-8 EBV virus in humanized mice (30), but other viruses such as the M81 EBV strain are more prone to lytic replication (31, 32). In contrast to these IM-like infections, lower viral doses might mimic asymptomatic primary infection, as it occurs in the vast majority of humans (6), but these have so far not extensively been explored in humanized mice (33). Nevertheless, all EBV infection programs can be found upon usually intraperitoneal inoculation of humanized mice.

According to the predominance of EBV latency III infection the virus-associated lymphomas that develop in up to one third of infected mice also carry this strongly growth transforming gene expression program akin to some diffuse large B cell lymphomas (DLBCL) and *in vitro* EBV transformed, B cell derived lymphoblastoid cell lines (LCLs) (2). Burkitt’s and Hodgkin’s lymphomas that carry latency I and II have so far not been reported. DLBCL-like lymphomagenesis can further be increased by infection with an EBV strain that is deficient in the viral tumor suppressor EBNA3B (34). Accordingly, EBNA3B loss has also be found in a subset of DLBCLs in human patients (34, 35). Furthermore, both EBV and KSHV are present in most PELs (36). Of all KSHV associated malignancies PEL cells are also the only one that maintains KSHV in culture (37). Interestingly, KSHV infection only persists in humanized mice upon co-infection with EBV (23, 38). Similarly, both viruses or their monkey orthologues seem to be co-transmitted in both macaques and humans (39, 40). Furthermore, EBV supports KSHV infection of B cells *in vitro* (41). KSHV and EBV co-infection of humanized mice also allows for the development of PEL-like lymphomas (Figure 1) with characteristics of plasma cell differentiation that have previously been defined for PELs (23, 42). Consistent with lytic EBV replication being associated with plasma cell differentiation (29) both PEL-like tumors in humanized mice and human PELs displayed elevated lytic viral gene products and this lytic EBV replication contributed to enhanced tumor formation in double-infected humanized mice (23). Thus, EBV associated DLBCLs and KSHV as well as EBV positive PELs can be elicited in humanized mice.

## Immune Control by Innate Leukocytes

The symptomatic primary EBV infection IM is an immunopathology caused by massive expansion of CD8^+^ T cells that are predominantly directed against lytic EBV antigens (43). This disease is rare in children but can make up 30 to 50% of primary infections in adolescents (6). Therefore, EBV infection at young age seems to trigger a different immune response than later in life. One reason for this altered immune response could be more robust innate immune control of lytic EBV infection in children. Along these lines it has been shown that NK cells protect from lytic EBV infection in humanized mice (19, 44) (Figure 1). In both humanized mice and pediatric IM patients primarily early differentiated CD56^dim^NKG2A^+^killer immunolglobulin-like receptor (KIR)^–^ NK cells expand (19, 45–48). While these are the most abundant NK cell subpopulation after birth in humans, they differentiate to CD56^dim^NKG2A^–^KIR^+^ NK cells during the first decade of life (45), possibly rendering adolescents more susceptible to IM. It has been suggested that ligands for the activating NK cell receptors NKG2D and DNAM1 get up-regulated on B cells that undergo lytic EBV reactivation, and that NKG2D and DNAM1 mediate NK cell cytotoxicity against the respective targets (49). Thus, NK cell differentiation could diminish protective NK cell populations during the first decade of life, compromising innate immune control of lytic EBV infection which in turn would trigger the massive expansion of lytic EBV antigen specific CD8^+^ T cells, causing IM.

However, in addition to NK cells other innate lymphocyte populations might play a role during EBV infection. Along these lines it has been shown that Vγ9Vδ2 T cells that recognize mevalonate metabolism intermediates presented by butyrophilin 2A1 (BTN2A1) prevent EBV associated lymphoproliferative disease in humanized mice (50). Similarly, the transfer of Vγ9Vδ2 T cells inhibited tumor growth in EBV transformed B cell carrying mice (51). Vγ9Vδ2 T cells recognized EBV infected B cells by their T cell receptor and NKG2D as a co-receptor (50). Especially EBV latency I Burkitt’s lymphoma cell lines expanded human Vγ9Vδ2 T cells (52). These lymphoma cell lines displayed the highest levels of mevalonate metabolite presentations on BTN2A1. Interestingly, only around 50% of human individuals could raise these protective Vγ9Vδ2 T cell responses against EBV infected targets (52). These so-called group 1 individuals harbor a high frequency of Vγ9Vδ2 T cells with T cell receptors that utilize JγP segments (53). These Vγ9JγP T cells expanded upon stimulation with Burkitt’s lymphoma cell lines. Thus, Vγ9Vδ2 T cells might protect preferentially against EBV latency I infected B cells.

Finally, there might also be a role for NKT cells that recognize glycolipid presentation on CD1d non-classical MHC class I molecules (54, 55). Adoptive transfer of NKT cells into mice that carried EBV transformed B cells reduced their tumor formation (54). They seemed to recognize freshly EBV infected B cells and EBV latency II infected tumor cells, while CD1d is down-regulated on EBV latency III infected B cells (55). Along these lines, X-linked lymphoproliferative (XLP-1) disease which affects primarily boys due to mutations in the 2B4 adaptor SAP and which confers life-threatening susceptibility to EBV associated pathogenesis, lack NKT cells (56). Thus, NKT cells might also contribute to innate immune control of latently EBV infected B cells, possibly preferentially expressing the latency II program. Humanized mice have contributed to demonstrate immune control by NK, Vγ9Vδ2 T cells, and NKT cells against lytic and latent EBV infected B cells, respectively. Only fully transformed EBV latency III B cells, resembling DLBCLs, do not seem to be efficiently recognized by innate lymphocytes, but these seem to target EBV infected precursors that are required for DLBCL-like tumor development in humanized mice (Figure 1).

## Cytotoxic T Cell Responses

In contrast to innate lymphocytes, EBV latency III infected B cells that can be also generated *in vitro* by EBV infection of human B cells, are highly immunogenic for T cells. Indeed, adoptive transfer of LCL stimulated T cell lines into patients with EBV induced post-transplant lymphoproliferative disease (PTLD) was one of the first cellular therapies and was able to cause PTLD regression (57). The EBV antigens EBNA1, LMP1, and LMP2 have proven to be sufficient as T cell targets for such adoptive T cell transfer therapies against EBV associated malignancies (58, 59). Similarly in humanized mice antibody mediated T cell depletion increased EBV viral loads and DLBCL-like lymphomagenesis (16, 60) (Figure 1). While primarily CD8^+^ T cells seemed to protect from EBV induced lymphomagenesis in humanized mice, antibody depletion, pharmacological inhibition by FK506 or destruction of CD4^+^ T cells by HIV co-infection also compromised EBV specific immune control in humanized mice (16, 21, 25). Along these lines, late lytic EBV antigen specific CD4^+^ T cells have been reported to restrict EBV transformed B cell growth in mice (61). Thus, both cytotoxic CD8^+^ T cells and helper T cell functions seem to be required for adaptive immune control of EBV in humanized mice.

Antibody blocking and recombinant EBV mutant infection experiments have revealed crucial aspects of this T cell mediated immune control of EBV in humanized mice and helped to explain the mechanistic underpinnings of EBV susceptibility in patients with the above mentioned primary immunodeficiencies. Among the co-stimulatory molecules, blocking of 2B4, one of the receptors that depends on SAP for activating signaling and is affected in XLP-1, led to increased EBV loads and lymphomagenesis (62). This 2B4 requirement was mainly on CD8^+^ T cells because their antibody mediated depletion did not further increase viral titers and tumor formation. In addition, CD8^+^ T cells with the inhibitory co-receptor PD-1 expanded during IM and in EBV infected humanized mice (33, 63). They included both latent (LMP2) and lytic (BMLF1) EBV antigen specific CD8^+^ T cells. These PD-1 positive CD8^+^ T cells carried the highest cytotoxic ability during EBV infection of humanized mice. Furthermore, they expressed CXCR5, Tim-3, KLRG1, and TCF-1 (33) and resembled germinal center homing CD8^+^ T cells which have previously been described to contain EBV specific CD8^+^ T cells in human tonsils (64). Interestingly, antibody blocking of PD-1 led to increased EBV viral loads and lymphomagenesis. This was associated with IL-10 production and might therefore represent activation of a regulatory CD4^+^ T cell population during PD-1 inhibition that compromises EBV specific immune control. Along these lines, at least a subset of patients that undergo PD-1 blockade for tumor treatment and develop neurological side effects, have recently been shown to harbor elevated EBV loads and accumulate EBV infected B cells and EBV reactive CD4^+^ and CD8^+^ T cells in their central nervous system (65). In contrast to PD-1 blockade on regulatory CD4^+^ T cells, humanized mouse models that sustained only T and EBV transformed B cell reconstitution developed exhausted, presumably CD8^+^ T cell populations and antibody blocking of their PD-1 and CTLA-4 inhibitory receptors restored EBV specific immune control (66). Antibody blocking studies have, therefore, suggested essential roles for PD-1, CTLA-4, and 2B4 during EBV specific immune control in humanized mice.

Additional characteristics of protective EBV specific T cell responses have been revealed by recombinant EBV mutant viruses. In addition to secondary lymphoid tissue homing, CXCR3 dependent T cell homing to inflamed tissues has been found to be critical for immune control of EBV in humanized mice by studying EBNA3B deficiency (34). EBNA3B deficiency which was observed in some patients with DLBCL (34, 35), elicited elevated lymphomagenesis in humanized mice (34). The respective tumors were devoid of T cell infiltrates and EBNA3B deficient LCLs were found to produce less of the CXCR3 stimulating CXCL9 and 10 chemokines. Transgenic CXCL10 expression by EBNA3B deficient LCLs restored their T cell mediated immune control *in vivo*. In addition, compromised MHC class I antigen presentation by EBV miRNAs limits EBV specific immune control (67). miRNA deficient EBV did not induce any DBLCL-like lymphomas in humanized mice, but tumor formation was restored upon antibody mediated CD8^+^ T cell depletion. Both latent and at least early lytic CD8^+^ T cell responses might contribute to CD8^+^ T cell mediated immune protection against EBV associated lymphomagenesis in humanized mice (18, 30). EBV deficient in the immediate early lytic EBV transactivator BZLF1 was compromised in its ability to cause DLBCL-like lymphomas in humanized mice, KSHV co-infection increased lytic EBV replication and lymphomagenesis, and BMLF1 specific CD8^+^ T cells were able to transiently control lytic EBV infection after their adoptive transfer (18, 23, 30). These studies define CXCR3 dependent homing, targeting of early lytic EBV antigens and efficient MHC class I antigen presentation as additional characteristics of EBV specific immune control by T cells.

In contrast to CD8^+^ T cells, CD4^+^ T cells can, however, also mediate pro-tumorigenic effects. LMP1 deficient EBV required CD4^+^ T cells for DLBCL-like lymphoma formation in humanized mice (68). Furthermore, CD4^+^ T cells allowed EBV to access the Burkitt’s and Hodgkin’s lymphoma associated EBV latencies I and II in humanized mice, even so no tumors with these viral gene expression patterns have so far been observed in any humanized mouse model (24). Therefore, both anti- and pro-tumorigenic functions of T cells can be studied during EBV infection of humanized mice, and so far, CXCR3^+^CXCR5^+^2B4^+^CD8^+^ T cells have emerged as the main protective entity.

## Vaccination

Epstein Barr virus specific immune control is uniquely dependent on cell mediated immunity and cytotoxic lymphocytes, while individuals with deficient antibody responses do not suffer from EBV associated pathologies (9–11). This cytotoxic immune control of EBV can be recapitulated in humanized mice (14). Therefore, humanized mice should be well suited to test EBV specific vaccine candidates and develop vaccines that efficiently prime cytotoxic lymphocytes and are also required to stimulate other anti-tumor immune protection (69). Unfortunately, it has been quite frustrating to identify vaccine candidates that manage to induce such protective T cell responses. Antigen targeting to dendritic cells (DCs), recombinant viral vectors, and virus like particles (VLP) have been explored.

Even so EBNA1 specific T cells have been therapeutically used against PTLD (58) and this antigen is incorporated in many vaccine formulations (70–74), targeting of EBNA1 to DCs and uptake via their DEC-205 receptor has elicited only weak CD4^+^ T cell responses (75, 76). However, even in regular mice this vaccine formulation was even with a boost by a recombinant adenovirus encoding EBNA1 less efficient in inducing T cell responses than heterologous prime-boost vaccination with EBNA1 encoding adenovirus and modified vaccinia virus Ankara (MVA) (74). In contrast, an EBV derived VLP into which EBNA1 was incorporated as a tegument component elicited protective CD4^+^ T cell responses against EBV challenge in humanized mice (77). This VLP targets B cells as EBV does, and the abundance of these antigen presenting cells (APCs) in humanized mice could facilitate the priming of sufficient numbers of protective T cells. Similarly, yellow fever virus (YFV) specific vaccination that is based on the attenuated viral strain YFV17D required human DC expansion prior to YFV17D injection to prime significant T cell responses (78). Such expanded APC populations, as exemplified by the high constitutive B cell reconstitution in humanized mice and Flt3L mediated DC expansion might overcome the paucity in secondary lymphoid tissue development that most humanized mouse models suffer from (79). Interestingly, adoptive transfer of DC populations did not only increase these APC populations and allowed for more efficient induction of human cytomegalovirus (HCMV) specific immune responses in humanized mice, but also induced the formation of secondary lymphoid tissues like lymph nodes (80). These structures then did not only facilitate the priming of T cell, but also antibody responses (80). Thus, humanized mice allow for the evaluation of vaccines, but might require expansion of APC populations and secondary lymphoid tissues for them to meet naïve T cells for adaptive cell-mediated and possibly even humoral immune response induction.

## Conclusion and Outlook

Humanized mice hold the promise that human immune responses and their modulation by immunotherapies as well as vaccinations can be studied. However, at least humoral immune responses are severely attenuated in these mice and mucosal immune reconstitution is only rudimentary. As a result, only few pathogens have been shown to elicit protective immune responses in humanized mouse systems. As these models get further improved by introduction of human MHC and cytokine genes, as well as additional human organoids (15) immunocompetence will probably increase. However, already with the available models strongly T cell immunogenic pathogens like EBV and KSHV can be characterized for their infection, tumorigenesis, and cell-mediated immune control. Indeed, EBV is the pathogen that most often leads to even pathogenic CD8^+^ T cell expansions in humans, as in IM, and primary immunodeficiencies as well as therapeutic adoptive T cell transfer suggest that such cytotoxic lymphocyte responses are necessary and sufficient for its immune control (9, 43, 81). This suggests that EBV is well suited to interrogate the immunocompetence of cytotoxic lymphocyte compartments in humanized mice and should be able to reveal improvements of these preclinical *in vivo* models.

At the same time any manipulation of T cell mediated immune control, like immune checkpoint inhibition, can be assessed for its ability to improve or compromise EBV specific immune control, even if the respective treatments are developed for other tumors than EBV associated malignancies. The influence of these treatments on EBV infection should still be of interest because the vast majority of adult humans (>95%) are persistently infected with EBV (2) and, therefore, any immune modulation of anti-tumor T cell responses will also affect EBV specific immune control in the respective patients, as becomes apparent during immunosuppressive treatment after transplantation, increasing the risk for PTLD (7, 21). Thus, EBV infected humanized mice constitute an interesting preclinical *in vivo* system to test human immune modulators.

Finally, it remains difficult to construct humanized mice with tumors and long-term engraftment of diverse autologous human immune system components (15). However, the growth transforming ability of EBV allows for the generation of autologous tumor cells that can be genetically manipulated and afterward implanted into humanized mice that have been reconstituted with CD34^+^ HPCs from the same donor. Therefore, it is one of the few systems that currently allow studying tumor interaction with human immune system components in an autologously xenografted humanized mouse model. All these advantages should allow us to interrogate human immune responses and design vaccines as well as other immunotherapies to elicit them more comprehensively.

## Author Contributions

CM wrote the manuscript.

## Conflict of Interest

The authors declare that the research was conducted in the absence of any commercial or financial relationships that could be construed as a potential conflict of interest.
